# Soluble (Pro)renin Receptor Is Adversely Associated with Indices of Left Ventricular Structure and Function: The African-PREDICT Study

**DOI:** 10.3390/jcdd9050130

**Published:** 2022-04-25

**Authors:** Lebo F. Gafane-Matemane, Ruan Kruger, Johannes M. Van Rooyen, Philimon N. Gona, Aletta E. Schutte

**Affiliations:** 1 Hypertension in Africa Research Team (HART), North-West University, Potchefstroom 2520, South Africa; ruan.kruger@g.nwu.ac.za (R.K.); johannes.vanrooyen@nwu.ac.za (J.M.V.R.); a.schutte@unsw.edu.au (A.E.S.); 2 Medical Research Council: Research Unit for Hypertension and Cardiovascular Disease, North-West University, Potchefstroom 2520, South Africa; 3 College of Nursing & Health Sciences, University of Massachusetts Boston, 100 Morrissey Boulevard, Boston, MA 02125, USA; phil.gona@umb.edu; 4 School of Population Health, University of New South Wales, The George Institute for Global Health, Sydney 2052, Australia

**Keywords:** renin, soluble (pro)renin receptor, ethnicity, relative wall thickness, left ventricular mass index, left ventricular ejection fraction, stroke volume

## Abstract

This study aims to compare soluble (pro)renin receptor [s(P)RR] levels between black and white adults and to explore the associations of left ventricular (LV) structure and function with s(P)RR in the total and ethnicity-stratified groups. The study sample included 1172 apparently healthy black (*n* = 587) and white (*n* = 585) participants of the African-PREDICT study aged 20–30 years. Echocardiography was performed to determine relative wall thickness (RWT), LV mass index, LV ejection fraction and stroke volume index (SVi). s(P)RR was analyzed from serum samples, while plasma renin activity-surrogate (PRA-S) and eq angiotensin II were determined using the RAS™ Fingerprint. s(P)RR was higher in the white participants compared to the black participants (*p* < 0.001). In multivariable-adjusted linear regression analyses, we observed a positive association between RWT and s(P)RR (β = 0.141; *p* = 0.005) and negative associations of LV ejection fraction (β = −0.123; *p* = 0.016) and SVi (β = −0.144; *p* = 0.004) with s(P)RR only in white adults. Higher s(P)RR observed in white vs. black participants was associated with higher RWT and poorer LV function only in young white adults but not in their black counterparts. These results suggest that s(P)RR may contribute to LV remodeling and dysfunction in white populations due to its role in volume–pressure regulation and its proinflammatory as well as profibrotic effects.

## 1. Introduction

The renin angiotensin system (RAS) is the major hormonal system involved in the regulation of fluid balance and blood pressure (BP) [1]. Consequently, this system forms the hallmark of therapeutic interventions in the treatment of hypertension and cardiovascular disease (CVD) [2,3]. Activation of RAS is initiated by the release of the enzyme renin from the juxtaglomerular apparatus in response to low perfusion pressure, among others [4,5]. Renin cleaves circulating angiotensinogen to form angiotensin I (AngI), which is then converted to angiotensin II (AngII) by angiotensin-converting enzyme (ACE) [4,5]. AngII directly and indirectly contributes to pathophysiological mechanisms leading to cardiac damage and remodeling, such as left ventricular (LV) remodeling, hypertrophy and associated complications [6,7,8]. The mechanisms, which are mediated by activation of the AngII receptor type 1 (AT1R) include chronic elevation of BP, inflammation, oxidative stress, tissue growth and cell proliferation [9,10].

(Pro)renin receptor [(P)RR], with an affinity for both prorenin and active renin, is expressed in various organs, including cardiac myocytes, the kidney and brain [11,12,13]. When activated, (P)RR elicits proinflammatory and profibrotic effects through the AngII-AT1R axis and intracellular signaling pathways such as mitogen-activated protein kinase (MAPK) and extracellular signal-regulated kinase 1/2 (ERK1/2) [13,14]. In earlier studies on the role of (P)RR as a biomarker, it was shown that binding of prorenin to (P)RR resulted in activation of MAPK p44/42 and tissue-growth factor-β (TGF-β), leading to increased contractility, hypertrophy and fibrosis [11,15]. Soluble (P)RR [s(P)RR], a product of (P)RR cleavage, is adversely linked to lipid and glucose metabolism and BP in mice [16]. In human studies, patients with heart failure presented with higher plasma s(P)RR levels as compared to healthy controls [17]. Observations on (P)RR and s(P)RR in relation to target organ damage have prompted suggestions that blockade of renin receptors may serve as a target for tissue protection [5]. However, evidence on the use of s(P)RR as a clinical marker is inconclusive, possibly due to limited data in population studies, especially on circulating s(P)RR. Nguyen et al. [18] showed that s(P)RR differed by ethnicity, being lower in black (*n* = 9) as compared to white men (*n* = 10). For the s(P)RR comparison, the study included a small sample of black and white participants, highlighting the need for further studies in human populations to confirm the ethnic differences in s(P)RR. Ethnic differences are important when investigating RAS due to the known lower levels of classical RAS components in black populations as compared to their white counterparts, and the impact on response to some classes of antihypertensive drugs [19,20,21].

Previous studies that included data from the African Prospective study on Early Detection and Identification of Cardiovascular disease and HyperTension study (African-PREDICT study) [22] have identified some of the biomarkers that may contribute to early cardiac deterioration. These markers include some of the well-known RAS components (renin, AngII, aldosterone excess) and sodium excretion [23,24,25,26]. Exploring the possible contribution of s(P)RR to LV structure and function will add to the knowledge and identification of potential targets for therapeutic intervention, given the scarcity of human data on s(P)RR. This study therefore compared s(P)RR levels between apparently healthy young black and white adults and explored whether LV structure and function are associated with s(P)RR in the total and ethnicity-stratified populations. 

## 2. Materials and Methods

### 2.1. Study Design and Population

This study utilized data from participants of the African-PREDICT study. The study sample included 1202 young adults prospectively followed over time to identify novel and early markers of cardiovascular risk [22]. To be eligible, participants were required to be self-reported black or white men and women, aged 20–30 years, with screening office BP <140/90 mmHg. Pregnant or lactating women, individuals who were on chronic medication or those who had been previously diagnosed with a chronic health condition were excluded. The present substudy used cross-sectional baseline data from 1172 participants with complete data for s(P)RR and echocardiographic measurements.

All participants gave written informed consent. The African-PREDICT study was approved by the Health Research Ethics Committee of the North-West University (NWU-00001-12-A1) and complied with the Declaration of Helsinki criteria for human research. The study is registered on ClinicalTrials.gov (NCT03292094).

### 2.2. Demographic, Anthropometric and Physical Activity Measurements 

Participant’s age, sex, ethnicity, alcohol, tobacco and medication use, and family history were obtained using a demographic and health questionnaire. The socioeconomic status (SES) of each participant was obtained using the Kuppuswamy’s Socioeconomic Status Scale 2010 adapted to the South African context [27]. The socioeconomic score was calculated based on skill level, education and household income by using a point system from the scale.

All anthropometric measurements were performed using the International Standards for Anthropometric Assessment [28] and included weight (kg) (SECA electronic scales, SECA, Birmingham, UK), height (m) (SECA stadiometer, SECA, Birmingham, UK) and waist circumference (WC) (Holtain, Crymych, UK). Body mass index (BMI) was calculated using the standard weight (kg)/height (m^2^) calculation. Body surface area (BSA) (m^2^) was additionally calculated using the Mosteller formula [29].

The ActiHeart device (CamNtech Ltd., England, UK), which was worn for a maximum of 7 days, was used to calculate average daily activity-energy expenditure (AEE) [30].

### 2.3. Cardiovascular Measurements

Ambulatory BP data was collected over a 24 h period using CardXplore devices (MediTech, Budapest, Hungary), programmed to take recordings every 30 min during the day (0600 to 2200 h) and every hour during the night (22:00 to 06:00 h). The device was fitted to each participant at approximately the same time every day (late morning), using an appropriately sized cuff as specified by the manufacturer. The mean inflation rate for this study population was calculated as 88% (standard deviation ± 12.3).

A standard transthoracic echocardiography procedure was followed while each participant was in a partial left decubitus position with the head of the examining table moderately elevated. The General Electric Vivid E9 device (GE Vingmed Ultrasound A/S, Hearten, Norway) was used along with the 2.5 to 3.5 MHz phased-array transducer and a three-lead electrocardiograph for timing purposes. Standardized methods were employed to obtain high-quality recordings according to the latest guidelines of the American Society of Echocardiography. LV mass was indexed for BSA (LVMi) and end-diastolic volume (EDVi), end-systolic volume (ESVi), LV internal diameter and posterior wall thickness at diastole for body height and stroke volume for height to the power of 2.04 (SVi) [31,32,33].

### 2.4. Biological Sampling and Biochemical Analyses

Participants were required not to eat or drink anything except water overnight for at least 8 h prior to undertaking the research measurements. Blood samples were collected early in the morning by a qualified nurse. The samples were then prepared according to standardized protocols and stored at −80 °C until the time of analysis. Serum samples were analyzed for creatinine, C-reactive protein (CRP), total and low- and high-density lipoprotein cholesterol (TC, LDL-c, HDL-c), glucose and gamma-glutamyl transferase (GGT) (Cobas Integra 400plus, Roche, Basel, Switzerland). 

Components of the RAS including PRA-s, ACE-s (eq AngII/eq AngI) and eq AngII were analyzed using the RAS-Fingerprint^®^ (Attoquant Diagnostics, Vienna, Austria). Angiotensin peptides were quantified based on a liquid chromatography coupled to tandem mass spectrometry (LC-MS/MS) multiplex assay in equilibrated serum volumes of 350 μL. Following a solid-phase-based and internal-standard-controlled extraction procedure, the LC-MS/MS quantification was performed using highly specific and simultaneous multiple reaction monitoring (MRM) detection of endogenous angiotensin peptides and internal standards [34,35]. Equilibrium angiotensin levels were further used to calculate a surrogate marker of renin enzyme activity, PRA-s (eq AngI + eq AngII). We measured serum s(P)RR (Immuno-Biological Laboratories Co., Ltd. (IBL-Japan)) and plasma prorenin (Human Prorenin ELISA Kit (Biovendor Laboratorni Medicina, Karasek, Czech Republic)). Estimated glomerular filtration rate (eGFR) was calculated using the Chronic Kidney Disease Epidemiology (CKD-EPI) formula without the race factor [36,37]. 

Each participant self-collected a 24 h urine sample on a day that was convenient for them, as recommended by the Pan American Health Organization/World Health Organization (PAHO/WHO) [38]. Urine samples were aliquoted and placed in a −20 °C freezer until analyses. Urinary creatinine, sodium and potassium were then measured using ion-selective electrode potentiometry on the Cobas Integra^®^ 400 plus (Roche, Basel, Switzerland) and were then used to calculate the 24 h urinary sodium:potassium ratio (Na^+^/K^+^).

### 2.5. Statistical Analyses 

Data analysis was performed with Statistica v13.3 (TIBCO software, Palo Alto, CA, USA). Prior to statistical analyses, normality assumption for quantitative variables was assessed using the Kolmogorov–Smirnov test and visual inspection of histograms. Normally distributed data were reported as the mean and standard deviation, and natural-logarithmically transformed data were presented by the geometric mean with 5th and 95th percentiles. Means across quartiles of s(P)RR were compared using ANOVA, followed by post hoc analyses where indicated, to compare pairwise means between quartiles. Chi-squared tests were used to compare quartiles of s(P)RR with qualitative variables. Independent sample t-tests were used to compare black and white participants. Ethnicity-pooled and ethnicity-specific regression analyses were performed to account for the well-known ethnic heterogeneity in RAS activity [19,25]. Candidate independent variables included age, ethnicity (in ethnicity-pooled analysis), sex, waist circumference, socioeconomic score, 24 h diastolic blood pressure, eGFR, Na^+^/K^+^, glucose, LDL-c, CRP, smoking, alcohol use and AEE as independent variables. Variables considered for entry in the multivariable linear regression analysis were chosen based on clinical, exploratory bivariate analysis, and partial correlations. Linear relationships were summarized using Pearson correlation coefficients and linear regression slopes and 95% confidence intervals. The proportion of variation in s(P)RR accounted for by regression models was summarized using adjusted R^2^. A *p*-value < 0.05 was considered statistically significant.

## 3. Results

### 3.1. Descriptive and Linear Regression Analyses

#### 3.1.1. Characteristics of the Study Sample

Ethnicity-pooled quartiles for s(P)RR were <20.40, 20.4–22.69, 22.70–25.31, and ≥25.31 ng/mL for Quartiles 1, 2, 3 and 4, respectively. Table 1 presents the profile of the participants stratified according to quartiles of s(P)RR. The proportion of women (*p*-trend = 0.008) and black participants (*p*-trend < 0.001) decreased with increasing quartiles of s(P)RR. All measures of body composition increased with s(P)RR (all *p*-trend < 0.001). 24 h systolic and diastolic BP increased with increasing s(P)RR (*p*-trend ≤ 0.001), SBP: 114 mmHg in quartile 1 vs. 120 mmHg in quartile 4, *p* < 0.001). End diastolic and systolic volume indices increased with s(P)RR quartiles (both *p*-trend < 0.001). LV internal diameter and posterior wall thickness at diastole and left atrial diameter to aortic root ratio (LA/Ao ratio) linearly increased across quartiles s(P)RR (all *p*-trend ≤ 0.022). Components of RAS increased across s(P)RR quartiles (all *p*-trend ≤ 0.032), while 24 h urinary Na^+^/K^+^ and eGFR showed a decreasing trend across s(P)RR quartiles (both *p*-trend ≤ 0.035; eGFR: quartile 1 vs. quartile 4, *p* ≤ 0.001). Metabolic variables and all inflammatory markers increased across the s(P)RR quartiles, with significantly higher levels in quartile 4 as compared to quartile 1 (all *p*-trend < 0.001, quartile 1 vs. quartile 4, all *p* < 0.001). A measure of energy expenditure decreased across s(P)RR quartiles (*p*-trend < 0.001) and quartile 4 had lower levels as compared to quartile 1 (5.11 vs. 6.31; *p* < 0.001).

**Figure 1:**
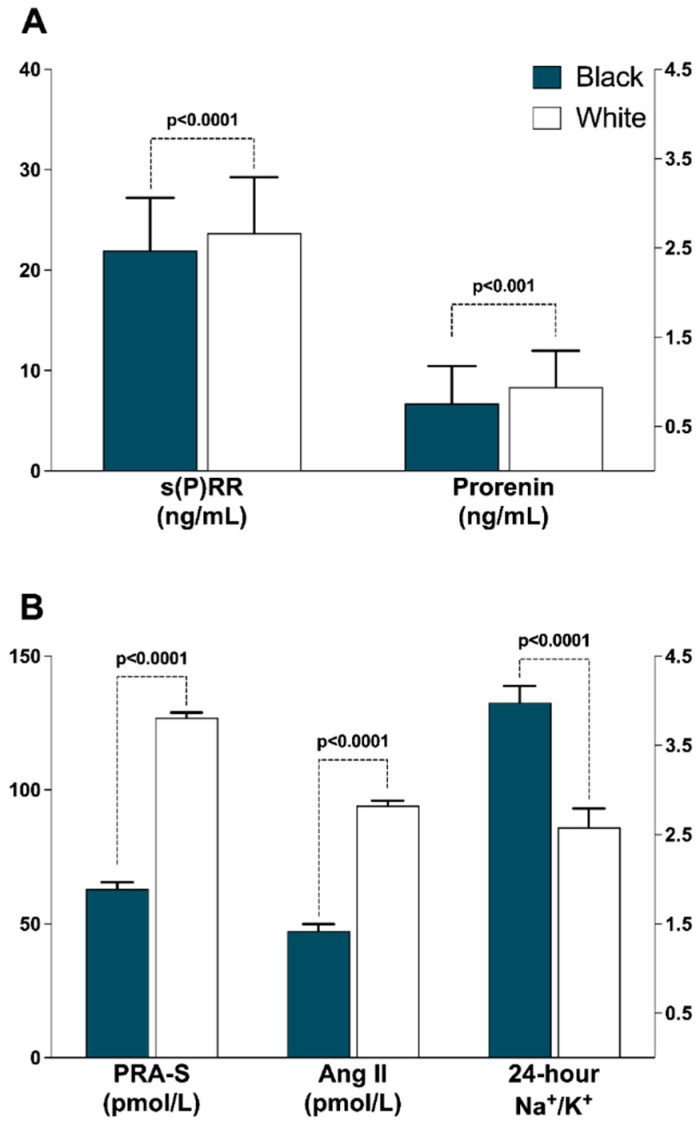
Ethnic comparisons of renin-angiotensin system profiles. (**A**) Soluble (pro)renin receptor and prorenin; (**B**) Plasma renin activity surrogate, Angiotensin II and Na^+^/K^+^. *Abbreviations:* s(P)RR, soluble (pro)renin receptor; PRA-S, angiotensin-based plasma renin activity, AngII, Angiotensin II.

Figure 1 represents differences in s(P)RR and other RAS components between black and white participants. s(P)RR, prorenin, PRA and eq AngII were higher in white participants as compared to their black counterparts (all *p* ≤ 0.001) and Na^+^/K^+^ was higher in the black compared to white participants (*p* < 0.001). Further comparisons between black and white participants are presented in Table 2. White participants had substantially higher values of measures of adiposity compared to their black counterparts (all *p* ≤ 0.005), whereas black participants had a greater proportion of women with waist circumference greater than the cut-off recommended by WHO in combination with the South African population-based guidelines (*p* < 0.001). In terms of echocardiographic data, the white participants had lower relative wall thickness; LV posterior wall thickness at diastole, ejection fraction and E/e’ ratio (all *p* ≤ 0.014); and higher LV internal diameter at diastole, end systolic and diastolic volume, stroke volume, E/A ratio and E/e’ ratio (all *p* ≤ 0.010).

#### 3.1.2. Linear Regression Analyses

**Figure 2:**
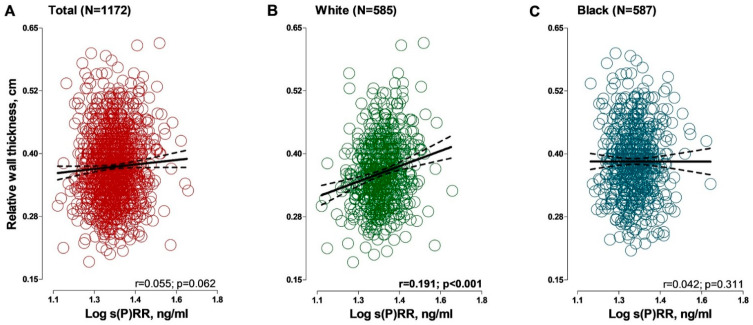
Associations between relative wall thickness and soluble (pro)renin receptor in (**A**) total group; (**B**) black group and (**C**) white group. Solid and dashed lines represent the regression line and 95% CI boundaries, respectively. *Abbreviations:* s(P)RR, soluble (pro)renin receptor.

In crude correlations (Figure 2, Table S1) a statistically significant but weak positive association was observed between LVMi (r = 0.063; *p* = 0.030) and s(P)RR in the sample including both black and white participants. A similar pattern was observed in the black participants for LVMi (r = 0.085; *p* = 0.039). SVi was associated positively with s(P)RR only in black participants (r = 0.146; *p* < 0.001). In white participants, relative wall thickness was positively associated with s(P)RR (r = 0.191; *p* = 0.001), which was not evident in black participants. LV ejection fraction showed a negative association with s(P)RR only in white participants (r = −0.138; *p* = 0.001). 

Analyses adjusted for sex, ethnicity and waist circumference in the total group (Table 3) revealed a statistically significant positive association between relative wall thickness and s(P)RR (r = 0.062; *p* = 0.036). In the participants as stratified by ethnicity, an additional adjustment was made for socioeconomic status score. In black participants, the positive association between SVi and s(P)RR persisted (r = 0.11; *p* = 0.008). In white participants, statistically significant partial-correlation coefficients were observed between relative wall thickness and LV ejection fraction and s(P)RR (all *p* ≤ 0.024), with an additional negative linear association observed between SVi and s(P)RR (r = −0.142; *p* = 0.001). 

In multivariable-adjusted linear regression analysis adjusted for ethnicity, sex, waist circumference, socioeconomic score and other covariates (Table 4), some of the associations between s(P)RR and measures of LV structure and function became attenuated and lost statistical significance, whereas new associations became evident. In ethnicity-pooled analysis, none of the associations remained statistically significant; however, a borderline-linear positive association was observed between s(P)RR and relative wall thickness (β = 0.073; *p* = 0.056). In white participants, there was a positive linear association between relative wall thickness and s(P)RR (β = 0.141; *p* = 0.005) and negative associations between LV ejection fraction (β = −0.123; *p* = 0.016) and SVi (β = −0.144; *p* = 0.004) and s(P)RR. The adjusted R-squared for LV ejection fraction was <0.10, but statistically significant (*p* < 0.001). Multivariable-adjusted linear regression models showing all covariates are shown in Tables S3–S5 and show s(P)RR as the main independent variables and the contribution of other covariates such as waist circumference, sex, ethnicity, glucose and Na/K to the models.

## 4. Discussion

This study investigated serum levels of s(P)RR in young black and white adults and associations with measures of LV structure and function due to the emerging role of s(P)RR in CVD and target organ damage. The main findings of the exploration were higher levels of s(P)RR in white as compared to black individuals and an independent positive association of relative wall thickness and s(P)RR, accompanied by negative associations of stroke volume and LV ejection fraction with s(P)RR only in white participants. To the best of our knowledge, this is the first investigation to show the difference in the presence of associations in a biethnic young population, with a relatively large sample size.

The higher levels of s(P)RR in the white population are consistent with a study by Nguyen et al., [18] which compared healthy men of black (*n* = 9) and white (*n* = 10) ethnicities aged 18–35 years. It was found that s(P)RR levels were lower in black men as compared to their white counterparts. Our study included apparently healthy black (*n* = 587) and white (*n* = 585) men and women with a comparable sex distribution and confirmed the influence of ethnicity on s(P)RR levels. As previously reported in this cohort [19], PRA-s, a measure of renin activity and eq AngII were two-fold higher in white participants as compared to black participants. Prorenin was higher in white participants which is inconsistent with recent relevant studies [18,41]. Tu et al. [41] found similar prorenin levels when comparing healthy black (*n* = 58) and white (*n* = 71) adults, a similar observation made by Nguyen et al. [18]. The discrepancies between the current study and previous observations could be partly due to the measurement technique and participant characteristics. In the current study, prorenin was measured directly in plasma, while in studies by Tu et al. [41] and Nguyen et al., [18], active renin was subtracted from total renin to obtain prorenin concentration values. Prorenin, when discovered, was initially regarded as an inactive precursor of the enzyme renin. However, data emerged showing that prorenin can bind with high affinity to (P)RR and result in various effects by activation of this receptor [11,42].

In the current study, BP and EDVi tended to increase as s(P)RR levels increased when investigated across s(P)RR quartiles, indicating involvement of s(P)RR in pressure and volume regulation. Kidney function (eGFR) declined, while glucose and LDL cholesterol increased across the s(P)RR quartiles. These observations represent the overall cardiovascular risk presented by high levels of s(P)RR, which may to some extent explain the adverse associations with LV structure and function. Previous observations have linked (P)RR polymorphisms to BP levels in Japanese men (mean age 61.1 ± 9.6 years) [43] and white men (mean age, 45.4 ± 14 years) [44], while in animals s(P)RR was positively associated with systolic BP [16]. Recently, Amari et al. [45] showed that s(P)RR is associated with cardiovascular events and mortality, serving as a biomarker to identify patients that require intensive care. The study population was older, with a number of CVDs and undergoing hemodialysis [45]. Our study already points to the usefulness of s(P)RR as a biomarker even in a healthy, young population (aged 20–30 years) with no current or historic CVD.

Previous studies have shown the detrimental effect of s(P)RR on kidney function and BP [46,47,48], with limited research on the role of s(P)RR in cardiac deterioration, particularly in young and healthy populations. To the best of our knowledge, the current study is the first to show the positive association between relative wall thickness in a healthy population, which may indicate the potential role of s(P)RR in early LV-structure alterations. The negative associations of two measures of LV function (LV ejection fraction and stroke volume) with s(P)RR further support its potential role in the early phases of LV remodeling and compromised functioning. The adverse association of LV structure and function were observed only in white participants and not in their black counterparts, which may be explained by the higher levels of s(P)RR and both its ligands, prorenin and active renin (represented by PRA) in white participants as compared to black participants. In Japanese women, a +1513A>G polymorphism of (P)RR was independently associated with the risk of LV hypertrophy and lacunar infarction, supporting the role of the renin receptor in target organ damage [49]. The mean age of women was 65.5 ± 5.6 years. When compared to men, 25.2% of the women presented with lacunar infarction, 13.5% presented with LV hypertrophy and 52.7% were classified as hypertensive [49]. In another study, plasma s(P)RR was found to be higher in patients with heart failure and associated with kidney damage in that group [17]. In our white participants with positive associations between relative wall thickness and s(P)RR, there was a comparable distribution of men and women, and sex was accounted for in multiple regression analyses. An important observation from our study was the substantially higher values of measures of adiposity in the white participants. This is despite a higher proportion of women with waist circumference greater than the cut-off point in the black participants, in which there were no statistically significant associations between s(P)RR and echocardiographic measures. The participants were normotensive at screening (clinic BP < 140/90 mmHg) and did not present with any known CVD. This may explain the absence of associations between s(P)RR and LVMi and only between relative wall thickness and s(P)RR in our study. Activation of s(P)RR by renin and/or renin can activate pathways via Ang II, leading to LV remodeling by promoting volume expansion and overload, inflammation and fibrosis as part of circulating RAS activation [50]. In addition to contributing to AngII formation, s(P)RR activation is involved in proinflammatory and profibrotic pathways that can set the stage for cardiac damage [11,15]. The absence of significant associations between LVMi and s(P)RR may be due to young age and healthy status and may be apparent when the study population is followed up over time with advancing age.

Our findings should be interpreted within the context of strengths and limitations. The cross-sectional nature of the investigation prevents us from making assumptions regarding the causal relationship between s(P)RR and markers of LV structure and function. The study sample was from one area of the North West Province of South Africa and cannot be regarded as representative of black and white populations in general. In the presence of these limitations, the current study confirmed some findings by earlier studies which included very small sample sizes and showed for the first time the positive association between relative wall thickness and s(P)RR in young healthy white participants.

In conclusion, we found higher levels of s(P)RR in healthy white young adults as compared to their black counterparts. s(P)RR was associated with an increase in relative wall thickness and adversely with LV function, an observation made only in white participants. These results suggest that s(P)RR may play a role in LV structural deterioration and compromised function in this study group. Our findings further support the notion that s(P)RR could serve as a cardiovascular risk marker, already in young populations without overt CVD; however, confirmation with follow-up data is required.

### Implications 

In this study we found higher levels of s(P)RR in young healthy white adults as compared to black adults. The independent positive association between s(P)RR and relative wall thickness, a marker of LV remodeling, accompanied by a negative association of s(P)RR with measures of LV function in a young healthy cohort without overt CVD suggest that s(P)RR may have the potential to serve as a biomarker of cardiovascular risk. Further research is required to understand the absence of relationships between s(P)RR and markers of LV remodeling in young black adults—a population known to be predisposed to hypertension-mediated organ damage and CVD.

## Figures and Tables

**Table 1:** Characteristics of the population across quartiles of soluble (pro)renin receptor.

	s(P)RR Q1 (*n* = 296) <20.40 ng/mL	s(P)RR Q2 (*n* = 290) 20.4–22.69 ng/mL	s(P)RR Q3 (*n* = 294) 22.70–25.31 ng/mL	s(P)RR Q4 (*n* = 292) ≥25.32 ng/mL	*p* Trend
**Sociodemographics**					
Age, years	24.7± 3.07	24.3 ± 3.08	24.3 ± 3.15	24.9 ± 3.13	0.032 *
Ethnicity/Black n (%)	200 (67.6)	156 (53.8)	131 (44.6)	100 (34.3)	**<0.001**
Sex/Female n (%)	172 (58.1)	151 (52.1)	158 (53.4)	129 (44.2)	**0.008**
Socioeconomic status					0.060
Low, n (%)	121 (40.9)	119 (41)	121 (41.2)	102 (34.9)	
Middle, n (%)	89 (30.1)	86 (29.7)	90 (30.6)	74 (25.3)	
High, n (%)	86 (29.1)	85 (29.3)	83 (28.2)	116 (39.7)	
**Anthropometric measurements**					
Waist circumference, cm	76.5 ± 9.70	78.0 ± 10.6	80.9 ± 12.6	84.9 ± 13.5	**<0.001 ^‡^**
Body weight, kg	66.1 ± 13.4	68.9 ± 15.4	72.0 ± 18.1	77.6 ± 18.4	**<0.001 ^‡^**
Body height, cm	167 ± 8.61	169 ± 9.45	168 ± 9.67	171 ± 9.89	**<0.001 ^‡^**
Body mass index, kg/m^2^	23.8 ± 4.56	24.2 ± 5.05	25.4 ± 5.42	26.8 ± 6.29	**<0.001 ^‡^**
**Blood Pressure and Echocardiographic variables**					
24 h systolic BP, mmHg	114 ± 9.50	116 ± 8.85	117 ± 9.30	120 ± 8.88	**<0.001 ^‡^**
24 h diastolic BP, mmHg	67.9 ± 5.97	68.0 ± 5.63	68.7 ±5.83	70.2 ± 5.89	**<0.001 ^‡^**
24 h heart rate, bpm	74.8 ± 10.3	73.4 ± 10.9	74.7 ± 9.85	74.7 ± 11.0	0.33
Relative wall thickness, cm	0.37 ± 0.07	0.37 ± 0.07	0.37 ± 0.07	0.38 ± 0.07	0.28
Interventricular septal thickness at diastole, cm	0.82 ± 0.17	0.82 ± 0.16	0.83 ± 0.17	0.84 ± 0.17	0.52
Interventricular septal thickness at systole, cm	1.07 ± 0.19	1.06 ± 0.18	1.08 ± 0.18	1.10 ± 0.18	0.10
LV internal diameter diastole, cm/m	2.74 ± 0.23	2.77 ± 0.23	2.81 ± 0.23	2.80 ± 0.25	0.002 *
LV posterior wall thickness diastole, cm/m	0.51 ± 0.08	0.51 ± 0.09	0.52 ± 0.08	0.53 ± 0.08	0.005 *
End systolic volume index, mL/m	19.5 ± 5.53	20.5 ± 5.59	22.1 ± 12.8	21.8 ± 7.17	<0.001 *
End diastolic volume index, mL/m	58.1 ± 12.1	60.1 ± 12.4	62.2 ± 13.2	62.9 ± 13.6	**<0.001**
Left ventricular mass index, g/m^2^	71.5 ± 18.0	73.2 ± 17.1	74.4 ± 16.7	74.6 ± 17.4	0.12
LV ejection fraction, %	66.3 ± 5.95	66.2 ± 5.81	65.6 ± 6.27	65.7 ± 6.26	0.36
Stroke volume index, mL/m^2.04^	23.2 ± 5.16	23.8 ± 5.25	24.2 ± 5.19	24.2 ± 5.45	0.055
E/A ratio	2.14 ± 0.55	2.22 ± 0.60	2.14 ± 0.57	2.08 ± 0.52	**0.029**
E/e’ ratio	6.35 ± 1.17	6.28 ± 1.14	6.39 ± 1.17	6.28 ± 1.13	0.59
LA/Ao ratio	1.06 ± 0.15	1.04 ± 0.15	1.07 ± 0.15	1.08 ± 0.14	**0.022**
**Kidney variables**					
Soluble (pro)renin receptor, ng/mL	18.4 (15.9–20.3)	21.6 (20.5–22.6)	23.9 (22.8–25.2)	28.4 (25.5–35.0)	**<0.001 ^‡^**
*^a^* Prorenin, ng/mL	0.71 (0.09–2.66)	0.92 (0.29–3.07)	0.84 (0.12–3.89)	0.90 (0.14–4.84)	0.013 *
Plasma renin activity-S, pmol/L	72.1 (13.1–269)	84.7 (13.9–272)	92.0 (15.1–303)	114 (23.1–373)	**<0.001 ^‡^**
eq Angiotensin II, pmol/L	54.7 (9.10–214)	62.9 (10.1–202)	68.9 (11.9–218)	83.2 (18.5–280)	**<0.001 ^‡^**
24 h urinary Na/K ratio	3.28 (1.24–7.62)	3.32 (1.45–7.03)	3.06 (1.21–6.69)	2.96 (1.08–6.44)	**0.035**
eGFR, mL/min/1.73 m^2^	124 ± 17.1	121 ± 19.3	120 ± 17.7	113 ± 19.0	**<0.001 ^‡^**
**Metabolic variables**					
Glucose, mmol/L	3.73 (2.37–5.39)	3.81(2.52–5.32)	3.89 (2.42–5.47)	4.39 (2.77–5.76)	**<0.001 ^‡^**
LDL-cholesterol, mmol/L	2.05 (0.99–3.81)	2.14 (0.98–3.74)	2.28 (1.10–4.26)	2.60 (1.36–4.53)	**<0.001 ^‡^**
HDL-cholesterol, mmol/L	1.08 (0.58–1.87)	1.08 (0.55–1.84)	1.08 (0.60–1.88)	1.10 (0.56–2.03)	0.92
C-reactive protein, mg/L	0.63 (0.05–5.85)	0.71 (0.09–9.08)	1.02 (0.12–8.58)	1.34 (0.11–13.7)	**<0.001 ^‡^**
Interleukin-6, mg/L	0.95 (0.33–2.91)	1.01 (0.38–3.98)	1.09 (0.40–3.70)	1.28 (0.47–4.38)	**<0.001 ^‡^**
Tumor Necrosis Factor-α, mg/L	0.91(0.34–2.41)	0.98 (0.38–2.09)	1.19 (0.62–2.84)	1.22 (0.60–2.66)	**<0.001 ^‡^**
Gamma-glutamyl transferase, U/L	16.5 (5.40–54.8)	16.7 (5.80–45.5)	18.3 (6.20–58.5)	21.9 (7.90–77.5)	**<0.001 ^‡^**
**Lifestyle factors**					
Self-reported smoking, *n* (%)	63 (21.5)	56 (19.3)	81 (27.6)	76 (26)	0.063
Self-reported alcohol use, *n* (%)	153 (52.4)	153 (52.8)	167 (57.2)	176 (60.7)	0.14
AEE, kCal/kg/day	6.31 ± 2.57	6.21 ± 3.16	6.0 ± 2.91	5.11 ± 2.79	**<0.001 ^‡^**

*^a^*—Data available for only 974 participants. Values are arithmetic mean ± standard deviation; geometric mean (5th and 95th percentile interval) for natural-logarithmically transformed variables. *Abbreviations:* LV, left ventricular; E/A ratio, peak early filling E-wave/late diastolic filling A-wave; E/e’ ratio, mitral peak velocity of early filling/early diastolic mitral annular velocity; LA/Ao ratio, Left atrial diameter to aortic root ratio; BP, blood pressure; eGFR, estimated glomerular filtration rate; LDL, low-density lipoprotein, HDL, high-density lipoprotein; AEE, activity-energy expenditure. Bold text indicates *p* < 0.05 across s(P)RR quartiles; ^‡^ *p* < 0.001, * *p* < 0.05 difference between Q1 and Q4.

**Table 2:** Comparisons between black and white adults.

	Black *n* = 587	White *n* = 585	*p*-Value
**Sociodemographics**			
Age, years	24.5 ± 3.17	24.6 ± 3.07	0.59
Sex/Female n (%)	303 (51.6))	307 (52.5)	0.77
Socioeconomic status			**<0.001**
Low, n (%)	345 (58.8)	118 (20.2)	
Middle, n (%)	161 (27.4))	178 (30.4)	
High, n (%)	81 (13.8)	289 (49.4))	
**Anthropometric measurements**			
Waist circumference, cm	77.8 ± 10.8	82.3 ± 12.9	**<0.001**
* Men, n (%)	77 (27.1)	83 (30.3)	0.41
* Women, n (%)	116 (38.3)	75 (24.4)	**<0.001**
Body weight, kg	66.2 ± 14.5	76.2 ± 17.8	**<0.001**
Body height, cm	164 ± 8.40	173 ± 8.79	**<0.001**
Body mass index, kg/m^2^	24.6 ± 5.70	25.5 ± 5.21	**0.005**
**Blood Pressure and Echocardiographic Variables**			
24 h systolic BP, mmHg	116 ± 8.99	118 ± 9.63	**0.001**
24 h diastolic BP, mmHg	68.8 ± 5.94	68.6 ± 5.86	**0.44**
24 h heart rate, bpm	75.3 ± 10.7	73.6 ± 10.3	**0.010**
Relative wall thickness, cm	0.38 ± 0.07	0.36 ± 0.07	**<0.001**
Interventricular septal thickness at diastole, cm	0.84 ± 0.17	0.82 ± 0.16	0.16
Interventricular septal thickness at systole, cm	1.08 ± 0.18	1.08 ± 0.18	0.71
LV internal diameter diastole/height, cm/m	2.76 ± 0.24	2.80 ± 0.24	**0.001**
LV posterior wall thickness diastole/height, cm/m	0.53 ± 0.08	0.50 ± 0.08	**<0.001**
End systolic volume index, mL/m	19.8 ± 9.72	22.1 ± 6.54	**<0.001**
Left ventricular mass index, g/m^2^	73.6 ± 18.4	73.2 ± 16.2	0.70
LV ejection fraction, %	66.4 ± 6.23	65.5 ± 5.89	**0.014**
Stroke volume index, mL/m^2.04^	23.5 ± 5.26	24.3 ± 5.27	**0.010**
End diastolic volume index, mL/m	58.0 ±11.9	63.9 ± 13.3	**<0.001**
E/A ratio	2.25 ± 0.61	2.04 ± 0.48	**<0.001**
E/e’ ratio	6.55 ± 1.18	6.10 ± 1.07	**<0.001**
LA/Ao ratio	1.06 ± 0.15	1.06 ± 0.15	0.45
**Kidney Variables**			
Soluble (pro)renin receptor, ng/ml	21.9 (19.9–29.2)	23.7 (17.9–31.6)	**<0.001**
Prorenin, ng/mL	0.76 (0.12–2.78)	0.94 (0.28–4.31)	**0.001**
Plasma renin activity-S, pmol/L	63.0 (11.4–263)	127 (39.0–337)	**<0.001**
eq Angiotensin II, pmol/L	47.3 (8.70–186)	94.1 (29.3–253)	**<0.001**
24 h urinary Sodium, mmol/L	114 (39.7–250)	102 (35.8–207)	**0.001**
24 h urinary Potassium, mmol/L	34.8 (13.4–101)	51.3 (22.8–112)	**<0.001**
24 h urinary Na/K ratio	3.98 (1.94–7.96)	2.58 (1.11–5.31)	**<0.001**
24 h urinary Creatinine, mmol/L	8.83 (3.02–21.7)	9.34 (4.03–20.9)	0.11
eGFR, mL/min/1.73 m^2^	123 ± 16.2	116 ± 20.4	**<0.001**
**Metabolic Variables**			
Glucose, mmol/L	3.79 (2.35–5.44)	4.11 (2.61–5.58)	**<0.001**
LDL-cholesterol, mmol/L	2.07 (0.99–3.70)	2.46 (1.23–4.42)	**<0.001**
HDL-cholesterol, mmol/L	1.08 (0.58–1.82)	1.09 (0.57–2.01)	0.78
C-reactive protein, mg/L	1.00 (0.10–10.1)	0.78 (0.08–8.08)	**0.003**
Interleukin-6, mg/L	1.24 (0.46–3.98)	0.93 (0.32–3.04)	**<0.001**
Tumor Necrosis Factor-α, mg/L	0.96 (0.37–2.43)	1.18 (0.59–2.51)	**<0.001**
Gamma-glutamyl transferase, U/L	22.3 (8.50–33.2)	14. 9 (5.40–47.0)	**<0.001**
**Lifestyle factors**			
Self-reported smoking, *n* (%)	147 (25.1)	129 (22.1)	0.22
Self-reported alcohol use, *n* (%)	325 (56.0))	324 (55.5)	0.85
ExpenditureAEE, kCal/kg/day	6.54 ± 2.91	5.38 ± 2.64	**<0.001**

Values are presented as arithmetic mean ± standard deviation; geometric mean (5th and 95th percentile interval) for natural-logarithmically transformed variables. * Waist circumference categories: WC > 94 cm for white men; >81.2 cm for black men; >80 cm for white women, and >81 cm for black women [39,40]. *Abbreviations:* BP, blood pressure; LV, left ventricular; E/A ratio, peak atrial early filling/late diastolic filling ratio; E/e’ ratio, mitral peak velocity during early filling/early diastolic mitral annular velocity ratio; LA/Ao ratio, left atrium diameter to aortic root ratio; eGFR, estimated glomerular filtration rate; LDL, low-density lipoprotein; HDL, high-density lipoprotein. Bold text indicates *p* < 0.05.

**Table 3:** Partial correlations between left ventricular indices and soluble (pro)renin receptor.

	Soluble (Pro)renin Receptor (ng/mL)		
	Total (*n* = 1172)	Black (*n* = 587)	White (*n* = 585)
Relative wall thickness, cm	**r = 0.062; *p* = 0.036**	r = −0.025; *p* = 0.552	**r = 0.154; *p* < 0.001**
Left ventricular mass index, g/m^2^	r = 0.018; *p* = 0.540	r = 0.078; *p* = 0.063	r = 0.042; *p* = 0.317
Left ventricular ejection fraction, %	r = −0.009; *p* = 0.765	r = 0.071; *p* = 0.085	**r = −0.094; *p* = 0.024**
Stroke volume index, ml/m^2.04^	r = −0.01; *p* = 0.659	**r = 0.11; *p* = 0.008**	**r = −0.142; *p* = 0.001**

Adjusted for ethnicity, sex and waist circumference. Additionally adjusted for socioeconomic score in the ethnicity-stratified participants.

**Table 4:** Associations between left ventricular structure and function and soluble (pro)renin receptor.

Dependent Variables	Soluble (Pro)renin Receptor (ng/mL)								
	Total (*n* = 1172)			Black (*n* = 587)			White (*n* = 585)		
	*^a^ R* ^2^	β (95% C.I.)	*p*	*^a^ R* ^2^	β (95% C.I.)	*p*	*R* ^2^	β (95% C.I.)	*p*
**Relative wall thickness, cm**	**0.075**	0.073 (−0.002; 0.138)	0.056	0.012	−0.001 (−0.12; 0.10)	0.86	**0.101**	**0.141 (0.039; 0.218)**	**0.005**
**Left ventricular mass index, g/m^2^**	**0.244**	0.037 (−7.13; 24.0)	0.29	**0.272**	0.081 (−3.91; 45.5)	0.102	0.213	−0.012 (−22.6; 17.33)	0.79
**Left ventricular ejection fraction, %**	**0.06**	−0.031 (−8.62; 3.55)	0.42	**0.049**	0.059 (−4.49; 14.6)	0.30	**0.072**	**−0.123 (−0.22; −0.023)**	**0.016**
**Stroke volume index, ml/m^2.04^**	**0.071**	−0.029 (−28.2; 1.91)	0.449	**0.104**	0.104 (−6.36; 37.6)	0.067	**0.247**	**−0.144 (−0.24; −0.045)**	**0.004**

*^a^*, Adjusted R^2^; Independent variables included in the multivariable-adjusted linear regression model: age, ethnicity (in total group), sex, waist circumference, socioeconomic score, 24 h diastolic blood pressure, estimated glomerular filtration rate, Na^+^/K^+^, glucose, low-density lipoprotein cholesterol, C-reactive protein, smoking, alcohol use and activity-energy expenditure. Bold value indicates statistical significance, *p* < 0.05.

## Data Availability

Data supporting the findings of this study are available upon request from the principal investigator (A.E.S.) upon reasonable request.

## Supplementary Materials

The following supporting information can be downloaded at: https://www.mdpi.com/article/10.3390/jcdd9050130/s1, Table S1: Pearson correlation coefficients between left ventricular indices and soluble (pro)renin receptor. Table S2: Multivariable-adjusted linear regression analysis of relative wall thickness with soluble (pro)renin receptor as main outcome variable. Table S3: Multivariable-adjusted linear regression analysis of left ventricular mass index with soluble (pro)renin receptor as main outcome variable. Table S4: Multivariable-adjusted linear regression analysis of left ventricular ejection fraction with soluble (pro)renin receptor as main outcome variable. Table S5: Multivariable-adjusted linear regression analysis of stroke volume with soluble (pro)renin receptor as main outcome variable.
