# Supplementary material for: Soluble (Pro)renin Receptor Is Adversely Associated with Indices of Left Ventricular Structure and Function: The African-PREDICT Study
Source: J Cardiovasc Dev Dis. 2022 Apr 25;9(5):130. doi: 10.3390/jcdd9050130 (PMC9144018; doi:10.3390/jcdd9050130)
Supplement: Supplementary file 1 [file jcdd-09-00130-s001.zip › jcdd-1655456-supplementary.pdf]

Journal of
Cardiovascular
Development and Disease

MDPI

Supplementary Tables

Soluble (Pro)renin Receptor Is Adversely Associated with Indices of Left Ventricular Structure and Function: The African-PREDICT Study

Lebo F. Gafane-Matemane [1,2,*], Ruan Kruger [1,2], Johannes M. Van Rooyen [1,2], Philimon N. Gona [3] and Aletta E. Schutte [1,2,4]

[1] Hypertension in Africa Research Team (HART), North-West University, South Africa, Potchefstroom; 2520; lebo.gafane@nwu.ac.za (L.F.G.-M.); ruan.kruger@g.nwu.ac.za (R.K.); johannes.vanrooyen@nwu.ac.za (J.M.VR.); a.schutte@unsw.edu.au (A.E.S.)
[2] Medical Research Council: Research Unit for Hypertension and Cardiovascular Disease, North-West University, South Africa, Potchefstroom, 2520
[3] College of Nursing & Health Sciences, University of Massachusetts Boston, 100 Morrissey Boulevard, Boston, MA 02125-3393, USA; phil.gona@umb.edu (P.N.G)
[4] School of Population Health, University of New South Wales; The George Institute for Global Health, Sydney 2052, Australia
* Correspondence: lebo.gafane@nwu.ac.za; Tel.: +27-18-299-2293

Table S1: Pearson correlation coefficients between left ventricular indices and soluble (pro)renin receptor

	Soluble (pro)renin receptor (ng/mL)		
	**Total (N=1172)**	**Black (N=587)**	**White (N=585)**
Relative wall thickness, cm	r= 0.055; p=0.062	r=0.042; p=0.311	**r=0.191; p<0.001**
Left ventricular mass index, g/m^2	**r=0.063; p=0.030**	**r=0.085; p=0.039**	r=0.049; p=0.240
Left ventricular ejection fraction, %	r=-0.055; p=0.059	r=0.055; p=0.184	**r=–0.138; p=0.001**
Stroke volume index, ml/m$^{2.04}$	r=0.055; p=0.058	**r=0.146; p<0.001**	r=–0.063; p=0.126

Table S2: Multivariable-adjusted linear regression analysis of relative wall thickness with soluble (pro)renin receptor as main outcome variable

**Independent variables**	**Relative wall thickness, cm**					
	**Total (N=1172)**		**Black (N=587)**		**White (N=585)**	
	**Adjusted R^2=0.075**		Adjusted R^2=0.018		**Adjusted R^2=0.101**	
	**βeta-coefficient**	***P***	**βeta-coefficient**	***P***	**βeta-coefficient**	***P***
Soluble (pro)renin receptor (ng/mL)	0.073	0.056	-0.010	0.87	**0.141**	**0.005**
Age, years	-0.053	0.187	-0.109	0.074	0.010	0.86
Ethnicity, Black/White	**-0.205**	**<0.001**	-	-	-	-
Sex, Male/Female	0.070	0.090	0.084	0.201	0.088	0.126
Socioeconomic Score	-0.009	0.83	0.013	0.83	-0.057	0.30
Waist circumference, cm	**0.119**	**0.006**	**0.172**	**0.009**	0.048	0.435
24-hour diastolic BP, mmHg	0.045	0.244	0.040	0.49	0.052	0.311
Estimated glomerular filtration rate, ml/min/1.73m^2	-0.074	0.099	-0.026	0.688	**-0.129**	**0.039**
Self-reported smoking, n (%)	0.032	0.382	-0.037	0.545	**0.097**	**0.042**
Self-reported alcohol use, n (%)	-0.029	0.340	0.015	0.790	-0.058	0.208
Activity Energy Expenditure, kCal/kg/day	-0.058	0.115	0.001	0.977	**-0.136**	**0.006**

24-hour urinary Na/K ratio	-0.019	0.627	0.010	0.859	-0.042	0.372
C-reactive protein, mg/L	-0.023	0.589	-0.034	0.616	-0.015	0.780
Glucose, mmol/L	**-0.182**	**<0.001**	**-0.144**	**0.027**	**-0.228**	**0.001**
Low density lipoprotein cholesterol, mmol/L	0.019	0.656	0.016	0.794	0.013	0.821

Independent variables included in the multivariable-adjusted linear regression model: age, ethnicity (in total group), sex, waist circumference, socioeconomic score, 24-hour diastolic blood pressure, estimated glomerular filtration rate, Na^+/K^+, glucose, low density lipoprotein cholesterol, C-reactive protein, smoking, alcohol use and activity energy expenditure. Bold value indicates statistical significance, $p<0.05$.

Table S3: Multivariable-adjusted linear regression analysis of left ventricular mass index with soluble (pro)renin receptor as main outcome variable

Independent variables	Left ventricular mass index, g/m2					
	Total (N=1172)		Black (N=587)		White (N=585)	
	Adjusted R^2=0.244		Adjusted R^2=0.27		Adjusted R^2=0.21	
	βeta-coefficient	*P*	βeta-coefficient	*P*	βeta-coefficient	*P*
Soluble (pro)renin receptor (ng/mL)	0.036	0.29	0.081	0.10	-0.012	0.79
Age, years	0.007	0.83	-0.011	0.83	0.022	0.68
Ethnicity, Black/White	-0.017	0.68	-	-	-	-
Sex, Male/Female	**0.450**	**<0.001**	**0.473**	**<0.001**	**0.427**	**<0.001**
Socioeconomic Score	-0.039	0.34	-0.020	0.70	-0.026	0.62
Waist circumference, cm	0.72	0.065	0.082	0.149	**0.113**	**0.048**
24-hour diastolic BP, mmHg	-0.032	0.35	0.033	0.51	**-0.104**	**0.032**
Estimated glomerular filtration rate, ml/min/1.73m^2	-0.073	0.071	-0.054	0.33	-0.086	0.14
Self-reported smoking, n (%)	0.017	0.604	-0.006	0.91	0.029	0.50
Self-reported alcohol use, n (%)	-0.015	0.64	-0.002	0.97	-0.033	0.44

Activity Energy Expenditure, kCal/kg/day	0.003	0.933	0.008	0.87	0.012	0.80
24-hour urinary Na/K ratio	-0.028	0.42	0.055	0.26	**-0.088**	**0.048**
C-reactive protein, mg/L	-0.056	0,14	-0.088	0.13	-0.008	0.878
Glucose, mmol/L	**-0.087**	**0.034**	-0.0608	0.28	-0.094	0.14
Low density lipoprotein cholesterol, mmol/L	-0.055	0.15	-0.049	0.35	-0.056	0.31

Independent variables included in the multivariable-adjusted linear regression model: age, ethnicity (in total group), sex, waist circumference, socioeconomic score, 24-hour diastolic blood pressure, estimated glomerular filtration rate, Na^+/K^+, glucose, low density lipoprotein cholesterol, C-reactive protein, smoking, alcohol use and activity energy expenditure. Bold value indicates statistical significance, $p<0.05$.

Table S4: Multivariable-adjusted linear regression analysis of left ventricular ejection fraction with soluble (pro)renin receptor as main outcome variable

**Independent variables**	**Left ventricular ejection fraction, %**					
	**Total (N=1172)**		**Black (N=587)**		**White (N=585)**	
	**Adjusted R^2=0.060**		Adjusted R^2=0.018		**Adjusted R^2=0.101**	
	**βeta-coefficient**	***P***	**βeta-coefficient**	***P***	**βeta-coefficient**	***P***
Soluble (pro)renin receptor (ng/mL)	-0.032	0.41	0.059	0.30	**-0.124**	**0.015**
Age, years	-0.050	0.21	-0.021	0.73	-0.096	0.097
Ethnicity, Black/White	-0.048	0.29	-	-	-	-
Sex, Male/Female	**-0.216**	**<0.001**	**-0.227**	**<0.001**	**-0.239**	**<0.001**
Socioeconomic Score	0.030	0.50	-0.024	0.68	0.092	0.099
Waist circumference, cm	-0.062	0.15	-0.082	0.20	-0.008	0.89
24-hour diastolic BP, mmHg	-0.038	0.31	-0.030	0.59	-0.028	0.60
Estimated glomerular filtration rate, ml/min/1.73m^2	-0.014	0.76	0.090	0.15	-0.108	0.16
Self-reported smoking, n (%)	0.032	0.39	0.061	0.31	0.017	0.73
Self-reported alcohol use, n (%)	0.056	0.11	0.063	0.25	0.007	0.88
Activity Energy Expenditure, kCal/kg/day	-0.035	0.34	-0.024	0.66	-0.056	0.27

24-hour urinary Na/K ratio	0.072	0.066	0.047	0.40	0.066	0.16
C-reactive protein, mg/L	0.073	0.083	0.104	0.11	0.044	0.41
Glucose, mmol/L	-0.002	0.96	-0.012	0.87	0.026	0.70
Low density lipoprotein cholesterol, mmol/L	0.036	0.39	0.062	0.307	-0.006	0.92

Independent variables included in the multivariable-adjusted linear regression model: age, ethnicity (in total group), sex, waist circumference, socioeconomic score, 24-hour diastolic blood pressure, estimated glomerular filtration rate, Na^+/K^+, glucose, low density lipoprotein cholesterol, C-reactive protein, smoking, alcohol use and activity energy expenditure. Bold value indicates statistical significance, $p<0.05$.

Table S5: Multivariable-adjusted linear regression analysis of stroke volume with soluble (pro)renin receptor as main outcome variable

**Independent variables**	**Stroke volume index, ml/m$^{2.04}$**					
	**Total (N=1172)**		**Black (N=587)**		**White (N=585)**	
	**Adjusted R^2=0.075**		**Adjusted R^2=0.104**		**Adjusted R^2=0.256**	
	**βeta-coefficient**	***P***	**βeta-coefficient**	***P***	**βeta-coefficient**	***P***
Soluble (pro)renin receptor (ng/mL)	-0.060	0.081	0.076	0.16	**-0.163**	**0.0003**
Age, years	0.019	0.60	0.039	0.50	0.012	0.81
Ethnicity, Black/White	**0.249**	**<0.001**	-	-	-	-
Sex, Male/Female	**0.282**	**<0.001**	**0.298**	**<0.001**	**0.24**	**<0.001**
Socioeconomic Score	-0.018	0.66	-0.012	0.83	-0.005	0.92
Waist circumference, cm	**0.281**	**<0.001**	**0.183**	**0.004**	**0.407**	**<0.001**
24-hour diastolic BP, mmHg	-0.064	0.061	0.008	0.898	**-0.12**	**0.010**
Estimated glomerular filtration rate, ml/min/1.73m^2	-0.027	0.507	0.003	0.97	-0.045	0.42
Self-reported smoking, n (%)	-0.017	0.62	0.048	0.409	-0.063	0.15
Self-reported alcohol use, n (%)	0.018	0.57	0.002	0.97	0.006	0.89
Activity Energy Expenditure, kCal/kg/day	0.028	0.418	0.023	0.66	0.034	0.45

24-hour urinary Na/K ratio	0.032	0.36	0.038	0.48	0.031	0.46
C-reactive protein, mg/L	0.034	0.37	0.046	0.47	0.038	0.43
Glucose, mmol/L	**0.118**	**0.004**	0.109	0.078	**0.149**	**0.015**
Low density lipoprotein cholesterol, mmol/L	-0.045	0.24	-0.019	0.74	-0.087	0.11

Independent variables included in the multivariable-adjusted linear regression model: age, ethnicity (in total group), sex, waist circumference, socioeconomic score, 24-hour diastolic blood pressure, estimated glomerular filtration rate, Na^+/K^+, glucose, low density lipoprotein cholesterol, C-reactive protein, smoking, alcohol use and activity energy expenditure. Bold value indicates statistical significance, $p<0.05$.
